# An examination of anxiety and its influence on health-related quality of life in Parkinson’s disease using the geriatric anxiety scale: a cross-sectional study

**DOI:** 10.1186/s12877-024-04911-8

**Published:** 2024-03-28

**Authors:** Konstantin G. Heimrich, Aline Schönenberg, Sarah Mendorf, Juliane Moussaoui, Tino Prell

**Affiliations:** 1 Department of Neurology, Jena University Hospital, Am Klinikum 1, 07747 Jena, Germany; 2 Department of Geriatrics, Halle University Hospital, Ernst-Grube-Straße 40, 06120 Halle, Germany; 3 Department of Trauma, Hand and Reconstructive Surgery, Jena University Hospital, Am Klinikum 1, 07747 Jena, Germany

**Keywords:** Parkinson´s disease, Anxiety, Quality of life, Depression, older adults

## Abstract

**Background:**

Anxiety is one of the most common but often overlooked mood-related nonmotor symptoms in people with Parkinson’s disease (PD). To improve the well-being of people with PD, it is important to understand the impact of anxiety in PD, especially its association with depressive and motor symptoms and its impact on health-related quality of life (HRQoL).

**Methods:**

91 people with PD were assessed between June 2017 and June 2018. Anxiety was measured using the Geriatric Anxiety Scale (GAS) and its cognitive, somatic, and affective subscales. HRQoL was assessed using the Parkinson’s Disease Questionnaire 39 (PDQ-39). Moreover, sociodemographic information, depressive symptoms, cognition, motor and nonmotor symptoms were assessed. Descriptive statistics, regression analyses, and path analyses were performed to understand predictors of anxiety and its influence on HRQoL.

**Results:**

Of the 91 people with PD, 35 (38.5%) experienced anxiety. Anxiety symptoms in these individuals primarily manifest as somatic sensations. Anxiety, motor, and depressive symptoms are interlinked but contribute individually to HRQoL. Beyond motor symptoms, cognitive and affective aspects of anxiety impact HRQoL. While anxiety and depression overlap, the somatic and cognitive aspects of anxiety play a significant role in determining HRQoL in addition to depressive symptoms.

**Conclusion:**

Our study used the GAS and its three subscales to shed light on the connections between anxiety, depression, and motor impairment in people with PD. Although anxiety is linked to depression and motor symptoms, it independently affects the HRQoL of people with PD. Thus, it is crucial to adopt a comprehensive diagnostic approach that detects and considers the impact of anxiety on HRQoL in PD.

**Supplementary Information:**

The online version contains supplementary material available at 10.1186/s12877-024-04911-8.

## Background

With a prevalence rate of 1%, Parkinson’s disease (PD) is one of the most common neurological disorders among individuals above 65 years, and owing to its neurodegenerative nature, its prevalence and burden increase further with advancing age [1]. PD is characterized by both motor and various nonmotor symptoms [2], and research suggests that the impact of nonmotor symptoms on health-related quality of life (HRQoL) exceeds that of motor symptoms [3]. Anxiety is the most prevalent mood-related nonmotor symptom, with an estimated prevalence of 31% in people with PD (PwPD) [4]. In addition, anxiety symptoms have a strong impact on patient’s lifestyle and their HRQoL [5–8]. However, anxiety symptoms are often inadequately recognized in PwPD [9, 10].

While older adults may experience anxiety for reasons common to all age groups, there are risk factors specific to age. These include changes in health and frailty, a decline in physical and mental functioning, and dependence. They may also experience pain, fear of worsening symptoms, and side effects of medication. Changes in socioeconomic and social status, isolation, and loss, can also contribute to distress [11]. For PwPD, anxiety levels can be further heightened due to disease-related complications [10]. Many PwPD experience episodic or anticipatory anxiety when their medication wears off [12], fearing the public display of motor symptoms such as gait problems or dyskinesia [13]. This can lead to embarrassment, stigma, fear of falling, and overall insecurity [14, 15]. Additionally, the severity of the disease, postural instability, gait dysfunction, and dyskinesias [16], as well as being female, having motor fluctuations, and a history of anxiety disorder [17] are risk factors for anxiety in PwPD.

Assessment of anxiety in older age is difficult because many symptoms of anxiety can also occur as medication side effects or as symptoms of physical illness. Especially somatic symptoms need to be carefully assessed to differentiate between somatic anxiety symptoms and physical illness [18].

Likewise, the assessment of anxiety in PwPD is difficult due to the complex relationship between anxiety, motor, and other nonmotor symptoms in PD [14]. Anxiety involves symptoms which may overlap with autonomic dysfunction and other PD-related somatic symptoms [15, 19]. Accordingly, it is important to consider mood symptoms as anxiety measures that are less influenced by motor and other nonmotor symptoms of PD [9]. However, there is a close association in particular between anxiety and depression, which often co-occur in PwPD [14, 20]. Thus, for adequate treatment of anxiety in PwPD, it is important to disentangle anxiety in particular from depression and motor symptoms.

Anxiety is significantly associated with decreased HRQoL in PwPD, as revealed by regression analyses [7, 8]. However, the association between HRQoL, anxiety, and depression or motor function needs to be further evaluated taking into account there directional dependencies. Path analysis is a valuable method for gaining this insigth.

In previous studies, several anxiety scales have been used in PwPD, such as the Hospital Anxiety and Depression Scale (HADS-A) or the Beck Anxiety Inventory (BAI) [21]. However, these scales are not primarily designed to assess levels of anxiety in PD and therefore showed unsatisfactory results [13, 14, 21]. Subsequently, the Parkinson Anxiety Score (PAS) was developed to improve these psychometric limitations [22]. However, a shortcoming of the PAS is that it only assesses common presentations of anxiety and insufficiently considers situational anxiety, e.g. due to impaired activities of daily living and the risk of falling [15]. This emphasizes the need to take better account of age-specific aspects of anxiety in PD as part of a more holistic approach. To incorporate age-related anxiety symptoms, the Geriatric Anxiety Scale (GAS) was developed as a self-report tool specifically designed for use with older adults. It is based on the Diagnostic and Statistical Manual of Mental Disorders (DSM-IV), which were then assessed in older adults to narrow the scale down to the items most present in advancing age. It differentiates between somatic, cognitive, and affective domains of anxiety [23]. A German version of the GAS was developed through a translation and back-translation process [24]. To the best of our knowledge, so far there was no research using the GAS to assess anxiety in PwPD in particular.

Understanding the impact of clinically relevant anxiety symptoms in PD, their association with depressive and motor symptoms, and their impact on HRQoL is crucial for developing effective interventions to improve the overall well-being of PwPD. Furthermore, recognizing the importance of anxiety in PD will help healthcare professionals provide comprehensive care, ultimately leading to better health outcomes. Therefore, this study aimed to (1) describe anxiety in older PwPD using the GAS, (2) identify its associated factors with a focus on depression and motor symptoms, and (3) assess the impact of anxiety on HRQoL.

## Methods

### Study design

Data from PwPD who were treated in the PD Multimodal Complex Treatment (PD-MCT) [25] from June 2017 until June 2018 at the Department of Neurology of the University Hospital Jena, Germany, were collected. All PwPD fulfilled the clinical diagnostic criteria of the Movement Disorder Society (MDS) [26]. The exclusion criteria were: refusal to participate, severe cognitive impairment measured by the Mini-Mental State Examination (MMSE) < 16 [27], and treatment with deep brain stimulation as it can have a negative effect on cognitive function [28]. A total of 130 PwPD were screened, of which 39 were excluded from the study for the above-mentioned reasons. Finally, data from 91 PwPD were analyzed.

### Variables

Anxiety was assessed using the German GAS [29]. The long form of the GAS consists of 30 self-report items, of which the first 25 represent three common domains of anxiety symptoms (cognitive, somatic, and affective). Items 26 to 30 additionally inquire about common areas of worry as an extension of the questionnaire. Respondents indicated how often they experienced each symptom in the past week on a four-point Likert scale (not at all “0”, sometimes “1”, most of the time “2”, always “3”), meaning that higher values indicate higher levels of anxiety. Based on the validation study of the German version of the GAS, item 9 (“I had difficulties staying asleep”) was removed from the subsequent analyses [24]. Accordingly, we determined the GAS total score for anxiety as a sum score of the first 24 questionnaire items (items 1 through 25, with the exception of item 9), and differentiated into a somatic (8 items; sum of items 1, 2, 3, 8, 17, 21, 22, and 23; item 9 excluded), cognitive (8 items; sum of items 4, 5, 12, 16, 18, 19, 24, 25), and affective subscale (8 items; sum of items 6, 7, 10, 11, 13, 14, 15, 20). In addition, for descriptive statistics, a dichotomization of the GAS total score was conducted to differentiate in PwPD without (GAS total score 0–15 points) and with clinically relevant anxiety (GAS total score > 15 points) [24].

HRQoL was assessed using the Parkinson’s Disease Questionnaire 39 (PDQ-39), a self-rated questionnaire comprising of 39 items divided into eight subscales. The PDQ-39 summary index is calculated as the mean of the eight subscales and may represent a single value for assessing patients’ overall HRQoL, with higher values indicating worse HRQoL [30].

In addition, the following variables were assessed: Age, sex, Movement Disorder Society-sponsored revision of the Unified Parkinson’s Disease Rating Scale (MDS-UPDRS) III [31], the sum scores of the Non-Motor Symptoms Questionnaire (NMS-Q) [32], the Mini-Mental State Examination (MMSE) [27], and the revised version of the Beck Depression Inventory (BDI II) [33].

### Statistical analyses

Descriptive statistics were used to characterize the cohort. Data were checked for normality using the Shapiro–Wilk test. Results were reported as the median and interquartile range (IQR) for non-normally distributed continuous variables or numbers and percentages (%) for categorical variables. Correlations between different clinical parameters were tested using Spearman’s correlation r_s_, and considered low (|r|= 0.1), moderate (|r|= 0.3), or strong (|r|= 0.5) [34]. For group comparisons, Mann–Whitney U-tests were performed for non-normally distributed data and chi-square tests for nominal data. The effect sizes of the Mann–Whitney U-test were determined by the rank-biserial correlation r_B_ and chi-square test using the Phi coefficient. Multiple linear regression analyses with backward selection (likelihood ratio) were performed to identify factors associated with anxiety and HRQoL. For the regression analyses, autocorrelation and multicollinearity were excluded (|r| < 0.8). Linearity was assessed using the Box–Tidwell procedure. Outliers were identified by calculating the standard deviation of the studentized residuals (SD > 3) and leverages (> 0.2), and were subsequently excluded from further regression analyses. Path analysis via structural equation models (SEM) with three variables was performed based on the results to understand the association between HRQoL, anxiety and depression or motor symptoms using the R package *lavaan* [35]. Path analysis allows the quantification of the relationships between different variables in a model by including every relationship between the variables of interest. The models were specified as follows:

**Table:** 

*direct effect: PDQ-39 *∼ c*BDI II *mediator: GAS* ∼ a*BDI II * PDQ-39 *∼ b*GAS indirect effect: a*b total effect: c + (a*b)	*direct effect: PDQ-39 *∼ c*MDS-UPDRS III *mediator: GAS* ∼ a*MDS-UPDRS III * PDQ-39 *∼ b*GAS indirect effect: a*b total effect: c + (a*b)

The level of statistical significance for all tests was set at *p* <.05 (two-tailed). IBM SPSS Statistics version 27 and R version 4.3.0 were used for statistical analyses.

## Results

### Descriptive analyses

Descriptive statistics of the study population are shown in Table 1. Of the 91 PwPD, 37 (40.7%) were female and 54 (59.3%) were male. The median age of the patients was 73 years (IQR = 69–79 years). Most patients had moderate motor impairment (median MDS-UPDRS III: 26.5 points; IQR = 18–37), reported 11 nonmotor symptoms based on the NMS-Q sum score (IQR = 6–15), and a median MMSE sum score of 27 points (IQR = 26–29). The median BDI II sum score was 12 points (IQR = 6–17).

**Table 1:** Descriptive statistics of the study population

	study population	no anxiety	anxiety	*p*	r
	(*N* = 91)	(*N* = 56)	(*N* = 35)		
Age (y), median (IQR)	73 (69–79)	75 (70–78.8)	71 (69–79)	0.243	/
PDQ-39 summary index	26.8 (14.1–43.1)	18.5 (11.9–34.8)	45.3 (24.1–52.8)	< 0.001*	0.464
MDS-UPDRS part III	26.5 (18–37)	24.5 (15–34.8)	34 (21.5–53.5)	0.017*	0.282
NMS-Q	11 (6–15)	10 (6–15)	12.5 (7.3–15)	0.534	/
MMSE	27 (26–29)	28 (26–29)	27 (25–29)	0.671	/
BDI II	12 (6–17)	8 (5–13)	17.5 (13–22.5)	< 0.001*	0.566
Sex (female), N (%)	37 (40.7)	23 (41.1)	14 (40.0)	0.919	/
GAS total score#	12 (6–19)	8 (4–11)	20 (19–25)	< 0.001*	0.839
Somatic Subscale#	6 (3–8)	4 (2.3–5.8)	9 (8–11)	< 0.001*	0.760
My heart raced or beat strongly (item 1)	0 (0–1)	0 (0–0)	1 (0–1)	< 0.001*	0.456
My breath was short (item 2)	0 (0–1)	0 (0–1)	1 (0–1)	< 0.001*	0.398
I had an upset stomach (item 3)	0 (0–1)	0 (0–0)	0 (0–1)	0.033*	0.224
I had difficulty falling asleep (item 8)	1 (0–1)	1 (0–1)	1 (0–2)	< 0.001*	0.369
I had difficulty staying asleep (item 9)#	1 (1–2)	1 (0–2)	2 (1–3)	0.013*	0.260
I had a hard time sitting still (item 17)	0 (0–1)	0 (0–0)	1 (0–1)	< 0.001*	0.488
I felt tired (item 21)	1 (1–1)	1 (0–1)	2 (1–2)	< 0.001*	0.574
My muscles were tense (item 22)	1 (0–1)	1 (0–1)	1 (1–2)	< 0.001*	0.448
I had back pain, neck pain, or muscle cramps (item 23)	1 (1–2)	1 (0–2)	2 (1–3)	0.001*	0.340
Cognitive Subscale	3 (1–5)	2 (0–3)	6 (5–9)	< 0.001*	0.746
I felt like things were not real or like I was outside of myself (item 4)	0 (0–0)	0 (0–0)	0 (0–0)	0.011*	0.266
I felt like I was losing control (item 5)	0 (0–1)	0 (0–0)	1 (0–1)	< 0.001*	0.453
I had difficulty concentrating (item 12)	1 (0–1)	1 (0–1)	1 (1–2)	< 0.001*	0.473
I felt like I was in a daze (item 16)	0 (0–1)	0 (0–0)	1 (0–1)	< 0.001*	0.439
I worried too much (item 18)	1 (0–1)	0 (0–1)	2 (1–2)	< 0.001*	0.594
I could not control my worry (item 19)	0 (0–1)	0 (0–0)	1 (0–2)	< 0.001*	0.540
I felt like I had no control over my life (item 24)	0 (0–1)	0 (0–0)	1 (0–1)	< 0.001*	0.464
I felt like something terrible was going to happen to me (item 25)	0 (0–0)	0 (0–0)	0 (0–1)	< 0.001*	0.398
Affective Subscale	3 (1–5)	2 (0–3)	6 (4–9)	< 0.001*	0.732
I was afraid of being judged by others (item 6)	0 (0–1)	0 (0–0)	1 (0–1)	< 0.001*	0.512
I was afraid of being humiliated or embarrassed (item 7)	0 (0–0)	0 (0–0)	0 (0–1)	< 0.001*	0.458
I was irritable (item 10)	1 (0–1)	0 (0–1)	1 (1–1)	< 0.001*	0.474
I had outbursts of anger (item 11)	0 (0–0)	0 (0–0)	0 (0–1)	< 0.001*	0.379
I was easily startled or upset (item 13)	0 (0–1)	0 (0–0)	1 (1–1)	< 0.001*	0.633
I was less interested in doing something I typically enjoy (item 14)	1 (0–1)	0 (0–1)	1 (1–2)	< 0.001*	0.388
I felt detached or isolated from others (item 15)	0 (0–1)	0 (0–0)	1 (0–1)	< 0.001*	0.547
I felt restless, keyed up, or on edge (item 20)	1 (0–1)	0 (0–1)	1 (1–1)	< 0.001*	0.558

Values are given as median and interquartile range unless otherwise indicated. Categorical parameters are given as absolute values and percentages. For group comparisons, Mann–Whitney U-tests were performed for non-normally distributed ordinal data and chi-square tests for nominal data. The effect sizes (r) of the group differences were determined using the rank biserial correlation for the Mann–Whitney U-test and the Phi coefficient for the chi-square test. BDI II: Revised version of Beck Depression Inventory sum score; GAS: German version of the Geriatric Anxiety Scale total score; MDS-UPDRS III: Movement Disorder Society-sponsored revision of the Unified Parkinson’s Disease Rating Scale part III; MMSE: Mini-Mental State Examination sum score; N: number of participants; NMS-Q: Non-Motor Symptoms Questionnaire total score; PDQ-39: Parkinson’s Disease Questionnaire 39. # Item 9 removed based on the validation study of the GAS. Significant group differences are indicated by *p <.05

Using a cut-off of > 15 points for clinically meaningful anxiety measured by the GAS, 35 participants (38.5%) were classified into the ‘anxiety’ group. Group comparisons for participants above and below the cutoff are shown in Table 1. PwPD who were classified as anxious had higher motor impairments (*r*_B_ = 0.282; *p* =.017), worse HRQoL as measured by the PDQ-39 summary index (*r*_B_ = 0.464; *p* <.001), and more depressive symptoms (*r*_B_ = 0.566; *p* <.001).

Looking more closely at the GAS, PwPD most frequently reported anxiety symptoms related to the somatic subscale (items 8, 21, 22, and 21). No ceiling effects were present for the GAS total score or any of the subscales. However, floor effects (a score of 0) were found in 15 participants (16.5%) for the cognitive and 16 participants (17.6%) for the affective subscale (see Fig. S1).

### Factors associated with anxiety

Next, we aimed to determine the sociodemographic and health-related factors associated with anxiety in PwPD. Spearman correlations revealed that depressive symptoms (BDI II: *r*_*s*_ = 0.62; *p* <.001) and motor impairment (MDS-UPDRS III: *r*_*s*_ = 0.26; *p* =.026) were associated with a higher GAS total score (Table S1). We then entered age, sex, BDI II, MDS-UPDRS III, NMS-Q, and MMSE in a multiple linear regression analysis with backward selection to assess how these factors influence anxiety as measured by the GAS total score. In the final model, the MDS-UPDRS III (*ß* = 0.34, 95% CI [0.036; 0.211], *p* =.007) and BDI II (*ß* = 0.47, 95% CI [0.273; 0.855], *p* <.001) were significantly associated with the GAS (F(2,45) = 16.84, *p* <.001, adjusted R^2^ = 0.40) (Table 2).

**Table 2:** Factors associated with anxiety (GAS total score)

		B	SD	95% CI lb	95% CI ub	ß	t	*p*
Model 1	(Constant)	4.286	8.835	-13.556	22.128		0.485	0.630
	Age	-0.069	0.084	-0.238	0.101	-0.096	-0.820	0.417
	Sex	0.462	1.566	-2.700	3.625	0.035	0.295	0.769
	BDI II	0.557	0.145	0.264	0.850	0.466	3.845	< 0.001^*^
	MDS-UPDRS III	0.138	0.046	0.045	0.231	0.376	2.989	0.005^*^
	NMS-Q	0.164	0.139	-0.116	0.444	0.140	1.184	0.243
	MMSE	-0.008	0.227	-0.467	0.451	-0.004	-0.036	0.972
Model 5	(Constant)	1.658	1.796	-1.959	5.274		0.923	0.361
	BDI II	0.559	0.142	0.273	0.844	0.467	3.941	< 0.001^*^
	MDS-UPDRS III	0.123	0.043	0.036	0.211	0.336	2.839	0.007^*^

Values were obtained using multiple linear regression analysis with backward selection to identify factors associated with anxiety. Dependent variable: GAS total score. Independent variables: Age, sex, BDI II, MDS-UPDRS III, NMS-Q, and MMSE. BDI II: Revised version of Beck Depression Inventory; GAS: German version of the Geriatric Anxiety Scale; MDS-UPDRS III: Movement Disorder Society-sponsored revision of the Unified Parkinson’s Disease Rating Scale part III; MMSE: Mini-Mental State Examination; NMS-Q: Non-Motor Symptoms Questionnaire. B: Unstandardized regression coefficient. *ß*: Standardized regression coefficient. CI lb: Lower bound of the confidence interval. CI ub: upper bound of the confidence interval. Significance is indicated by *p <.05

### Association between anxiety and HRQoL

To understand the impact of anxiety in PwPD, we examined the association between anxiety and HRQoL as measured by the PDQ-39 summary index. In an initial linear model using the GAS total score (F(1,72) = 48.94, *p* <.001, adjusted R² = 0.40), the GAS explained 40% of the variance in the PDQ-39 summary index (*ß* = 0.64, 95% CI [0.90; 1.62], *p* <.001).

Next, age, sex, BDI II, MDS-UPDRS III, NMS-Q, MMSE, and GAS were entered in a multiple linear regression analysis with backward selection, with the PDQ-39 summary index as dependent variable. In the final model, only the MDS-UPDRS III (*ß* = 0.24, 95% CI [-0.004; 0.480], *p* =.054) and the GAS total score (*ß* = 0.58, 95% CI [0.921; 2.210], *p* <.001) remained (F(2,44) = 23.88, *p* <.001, adjusted R^2^ = 0.50) (Table 3).

**Table 3:** Factors associated with PDQ-39 summary index

		B	SD	95% CI lb	95% CI ub	ß	t	*p*
Model 1	(Constant)	-6.122	33.672	-74.230	61.987		-0.182	0.857
	Age	0.156	0.324	-0.499	0.811	0.059	0.481	0.633
	Sex	-3.354	4.277	-12.005	5.297	-0.093	-0.784	0.438
	BDI II	-0.001	0.382	-0.773	0.772	< 0.001	-0.002	0.998
	MDS-UPDRS III	0.239	0.134	-0.031	0.509	0.236	1.791	0.081
	NMS-Q	0.087	0.380	-0.680	0.855	0.027	0.231	0.819
	MMSE	-0.004	0.662	-1.343	1.335	-0.001	-0.006	0.995
	GAS	1.539	0.414	0.701	2.376	0.570	3.715	0.001^*^
Model 6	(Constant)	3.685	4.114	-4.605	11.976		0.896	0.375
	MDS-UPDRS III	0.238	0.120	-0.004	0.480	0.235	1.981	0.054
	GAS	1.565	0.320	0.921	2.210	0.580	4.894	< 0.001^*^

Values were obtained using multiple linear regression analysis with backward selection to identify predictors of HRQoL. Dependent variable: PDQ-39 summary index. Independent variables: Age, sex, BDI II, MDS-UPDRS part III, NMS-Q, MMSE, and GAS. BDI II: Revised version of Beck Depression Inventory; GAS: German version of the Geriatric Anxiety Scale; MDS-UPDRS III: Movement Disorder Society-sponsored revision of the Unified Parkinson’s Disease Rating Scale part III; MMSE: Mini-Mental State Examination; NMS-Q: Non-Motor Symptoms Questionnaire, PDQ-39: Parkinson’s Disease Questionnaire 39. B: Unstandardized regression coefficient. *ß*: Standardized regression coefficient. CI lb: Lower bound of the confidence interval. CI ub: upper bound of the confidence interval. Significance is indicated by *p <.05

Lastly, we aimed to determine how the GAS subscales were associated with HRQoL. Using linear regression with backward selection, we revealed that both the cognitive (*ß* = 0.31, 95% CI [0.120; 4.588], *p* =.039) and affective GAS subscales (*ß* = 0.29, 95% CI [0.145; 4.732], *p* =.038) were independently associated with worse HRQoL as measured by the PDQ-39 summary index (F(3,45) = 15.39, *p* <.001, adjusted R^2^ = 0.47), but not the somatic subscale. Instead, the MDS-UPDRS III (*ß* = 0.28, 95% CI [0.042; 0.518], *p* =.022) was significantly associated with HRQoL (Table S2).

### Disentangling anxiety, depression and motor symptoms

**Fig. 1:**
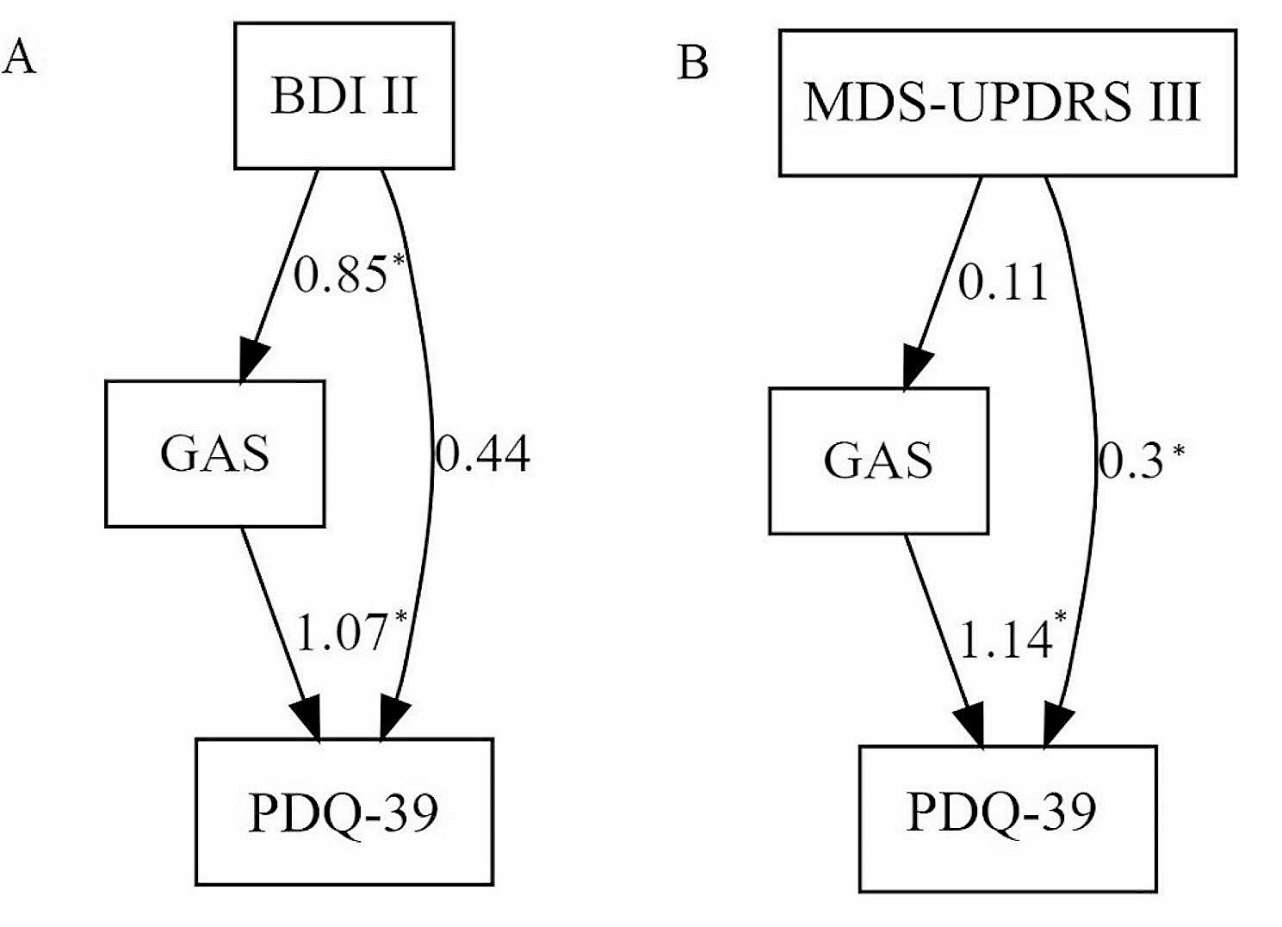
Path Analysis Models with path coefficients of the BDI II (**A**) and MDS-UPDRS III (**B**). BDI II: Revised version of Beck Depression Inventory sum score; GAS: German version of the Geriatric Anxiety Scale total score; MDS-UPDRS III: Movement Disorder Society-sponsored revision of the Unified Parkinson’s Disease Rating Scale part III; PDQ-39: Parkinson’s Disease Questionnaire 39. * indicating significant paths at a significance level of *p* <.05

As revealed by linear regression, the BDI II is significantly associated with the GAS (Table 2) and the PDQ-39 summary index (*p* <.001) in a univariate model (see Table S3 A). However, this association between HRQoL and BDI II disappeared (*p* =.163) when the GAS was added (*p* <.001) to the model (Table S3 A). Likewise, model comparison via ANOVA and common performance indices suggest that the BDI II itself does not contribute significantly to the explained variance of the PDQ-39 when the GAS is already present (Table S3 A). ANOVA revealed no difference between a model containing solely the GAS as a predictor of the PDQ-39, and a model containing both GAS and BDI II (*p* =.163). To disentangle the influence of the GAS and the BDI II on the PDQ-39, we performed path analysis with maximum likelihood estimator and Nonlinear Minimization subject to Box Constraints using the R-Package *Lavaan.* This approach (Fig. 1A) revealed an indirect effect of the BDI II on the PDQ-39 summary index (est = 1.07, *p* <.001) via the GAS (est = 0.85, *p* <.01), but no direct effect (est = 0.44, *p* =.149) of the BDI II on the PDQ-39 when the GAS is present.

To understand which aspects of the GAS contribute to the PDQ-39, we performed linear models for all three GAS subscales using the respective subscale and the BDI II as predictors (Table S3 A). The results suggest that the BDI II does not contribute significantly to the explained variance of the PDQ-39 summary index (*p* =.063) when the affective subscale (*p* =.024) is present. However, when considering only the somatic (*p* <.001) and the cognitive subscales (*p* <.001), the BDI II contributes significantly to the PDQ-39 (*p* =.001 and *p* =.040).

In contrast, using the same approach with the GAS and the MDS-UPDRS III as predictors of the PDQ-39 summary index revealed a continuous contribution of both variables to the explained variance of the PDQ-39 (Table S3 B). The GAS remained a significant predictor of the PDQ-39 (*p* <.001) even when the MDS-UPDRS III was added to the model (*p* =.001). Similarly, model comparison revealed that the model containing both variables was preferable (*p* <.001). Thus, a path analysis using the MDS-UPDRS III and the GAS (Fig. 1B) revealed a significant direct effect of the MDS-UPDRS III on the PDQ-39 summary index (est = 0.295, *p* <.001) but no indirect effect of the MDS-UPDRS III (est = 0.105, *p* =.65) via the GAS (est = 1.136, *p* <.001). When looking at the GAS subscales individually, all subscales contribute significantly to the PDQ-39 on top of the MDS-UPDRS III (*p* <.001) (Table S3 B).

## Discussion

The aim of the present study was to utilize the GAS in a population of PwPD and assess the predictors of anxiety in this particular patient population, as well as its impact on HRQoL. Additionally, we explored the relationship between anxiety and depression and between anxiety and motor symptoms as measured by the MDS-UPDRS III.

In our study, using a pre-defined cut-off of > 15 points in the GAS [24], 38.5% of patients were classified as anxious. This is in line with the prevalence range of 25 to 46% identified by a recent meta-analysis [4], with an average point prevalence of 31%. However, it must be noted that the prevalence rate in our data is based on a mometary self-reported assessment of anxiety symptoms and does not replace a thorough psychological assessment or indicate an anxiety disorder.

The most frequent anxiety symptoms in our cohort of patients with PwPD were somatic, mainly related to sleep and fatigue. Additionally, concentration and lack of joy were prevalent symptoms. This agrees with other studies indicating that in PwPD, anxiety often centers around the somatic display of symptoms [19]. The relationships between anxiety and concentration, sleep problems, fatigue, and depressive symptoms such as loss of joy have previously been identified, see Abou Kassm et al. (2021) for a review [14].

Using the GAS as well as medical and psychosocial covariates such as age, cognition, nonmotor symptoms, depressive symptoms measured by the BDI II, and motor symptoms assessed with the MDS-UPDRS III, we aimed to understand how anxiety impacts HRQoL. Using linear regression, our study confirmed anxiety as one of the main predictors of low HRQoL. This is in line with previous research highlighting anxiety as crucial for HRQoL in PwPD, exceeding the influence of motor symptoms [5, 6, 14]. With subscale analyses using the somatic, affective and cognitive subscales of the GAS, we identified that the cognitive and affective subscales contribute independently to worse HRQoL [14], whereas the somatic subscale does not when the MDS-UPDRS III is included in the model. In the next step, we utilized this relationship between HRQoL and the GAS subscales to analyze the relationship between GAS and both motor symptoms or depressive symptoms [14, 15, 19].

Regarding the separability of motor symptoms as assessed by the MDS-UPDRS III and anxiety symptoms as assessed by the GAS, our data revealed that both contribute independently to HRQoL. Using linear regression, we identified both GAS and MDS-UPDRS III as significant predictors for HRQoL. Likewise, using path analysis and model comparison, we revealed the best model fit for a model containing both variables, and no indirect effect of MDS-UPDRS III and GAS was found on HRQoL. These results suggest that there is some overlap between MDS-UPDRS III and GAS, which is confirmed by the fact that the MDS-UPDRS III is a predictor of the GAS [14], but that the GAS contains symptoms that go further than motor symptoms. This becomes evident when looking at the GAS subscales: while the somatic subscale of the GAS is most closely related to motor symptoms and is thus subordinate to the MDS-UPDRS III, the affective and cognitive subscales are not and contribute significantly to HRQoL above and beyond motor symptoms. This is in line with research by Rutten et al. [19] suggesting that especially affective symptoms are a reliable measure of anxiety in PwPD. While some studies suggest shared biological mechanisms behind anxiety and motor symptoms, it is also probable that the presence of symptoms lead to anxious feelings such as shame and fear of stigmatization, social anxiety, and fear of disease progression or complications [6, 10, 14].

While our results suggest that anxiety as measured by the GAS can be separated from motor symptoms assessed by the MDS-UPDRS III, the relationship between anxiety and depression is of equal interest. Along with the MDS-UPDRS III, depressive symptoms as assessed by the BDI II were identified as predictors of the GAS in our participants. The GAS and BDI II were highly correlated in our analysis, indicating that depressive symptomology and anxiety overlap. This overlap may partially be explained by similar items in both questionnaires, such as loss of interest, problems with concentration, fatigue, sleep problems, or loss of energy [19]. However, when assessing the influence on HRQoL, the BDI II relinquished its influence on HRQoL when the GAS was added to the model, indicating that the GAS covers aspects of the BDI II but contributes beyond those. Likewise, path analysis revealed that there is no direct effect of the BDI II on HRQoL when considering the GAS, meaning that GAS contributes on top of depressive symptoms. These results shed light on the ongoing debate regarding depression and anxiety by suggesting that despite a close relation, in the particular case of geriatric PwPD, the GAS captures aspects related to the HRQoL that the BDI II cannot. In line with a study by Rutten et al., our subscale analyses revealed that especially the affective subscale is closely related to depressive symptoms [19], whereas the somatic and cognitive subscales contribute beyond symptoms covered by the BDI II.

Generally, while our results indicate that different subscales of the GAS enable a separation of anxiety from depressive and motor symptoms, our study also confirms the complex interactive nature of all three constructs [14, 19]. Whereas the somatic and cognitive subscales serve as additional input to the BDI II when assessing HRQoL, the affective and cognitive subscales separate anxiety from motor symptoms. This link may be due to the diagnostic criteria for anxiety [36], which include both depressive and physical symptoms that strongly overlap with motor symptoms in PD, hindering a clear distinction [19].

Taken together, our study emphasizes the impact of anxiety in PwPD. Given its high prevalence and its negative effect on HRQoL, it is crucial to adopt a comprehensive diagnostic approach to screen for the presence of anxiety, determine the severity, and further assess the effect of beneficial treatment strategies [15]. Our study aimed to describe anxiety and its associated factors in particular in older PwPD. Previous research mainly focussed on the validation of known anxiety scores in PD or the development of more PD-specific anxiety rating scales (e.g., the PAS) [13, 22]. However, a shortcoming of the PAS is that it only assesses common presentations of anxiety [15]. In this regard, situational anxiety e.g. due to impaired activities of daily living and the risk of falling are insufficiently assessed [15, 37]. Given the high prevalence of impaired functional abilities in older age, it is crucial to consider age-specific aspects of anxiety in addition to PD-specific aspects for a more holistic approach. Accordingly, further research is required to develop and evaluate an appropriate PD-specific anxiety questionnaire that also covers inherent age-specific aspects of anxiety. Moreover, there is a need to further investigate whether existing evidence-based therapeutic options (e.g., cognitive behavioral therapy [38, 39], yoga [40, 41], acupuncture [42]) can also effectively reduce anxiety in older PwPD.

Our study has certain limitations. Our analyses are based on a selective group of PD patients who were included in a Multimodal Complex Treatment program. The small sample size and mono-centric data collection limit the generalizability of the obtained results. Due to the exclusion criteria, especially the exclusion of patients with severe cognitive impairment, the data generated are not fully representative of the PD population. As the included patients had high MMSE scores, our findings cannot be generalized to individuals with advanced cognitive decline. As we intended to identify associated factors of anxiety with a focus on depression and motor symptoms, we only used the MDS-UPDRS III to describe motor impairment and did not use other descriptions of disease severity (e.g., disease duration, Hoehn and Yahr stage, and levodopa equivalent daily dose). The analyzed anxiety and HRQoL measures were recorded based on self-reports and may have depended on mood and motivation. Although the utilization of self-report instruments may introduce bias, using patient-reported outcomes is essential when assessing personal experiences such as HRQoL and anxiety, and all questionnaires used are validated and commonly applied in scientific research. However, the relative influence of a variable on others depends strongly on the questionnaire used; thus, the data must be contextualized in comparison to other questionnaires. Likewise, the use of a scientific questionnaire only reflects momentary symptom assessments and does not replace a thorough diagnostic procedure to detect anxiety disorders. Additionally, fluctuations of anxiety (e.g., between the off- and on-medication states) [12], HRQoL and motor symptoms were not considered. Moreover, for descriptive statistics, clinically meaningful anxiety symptoms were defined as having a cut-off score > 15 points on the GAS. However, to the best of our knowledge, this is the first time the GAS has been used in PwPD and this cut-off needs to be evaluated in future studies. Furthermore, there are two limitations inherit to our regression approach: backward selection does not consider all possible combinations of potential predictors, which can sometimes lead to an unstable selection of variables, especially when the number of cases is relatively small. Lastly, cross-sectional data can neither consider causal relationships between variables, nor result in causal findings. Therefore, longitudinal data collection is needed in future studies to fully assess how the variables of interest are related. Longitudinal data is also required to distinguish between episodic and chronic anxiety. While SEM provides insight into the relationship between different variables when based on theoretical and empirical assumptions, causal relationships can only be confirmed using longitudinal data. The included patients were not diagnosed with anxiety disorders and showed relatively low scores on the GAS, therefore, the analysis should be repeated in patients with more severe levels of anxiety.

## Conclusion

Anxiety symptoms in PwPD are predominantly somatic. Somatic anxiety symptoms are closely related to motor symptoms as measured by the MDS-UPDRS III. However, the cognitive and affective aspects of anxiety contribute to HRQoL beyond motor symptoms. Regarding the overlap with depressive symptomology, both are closely related but the somatic and cognitive aspects of anxiety contribute to HRQoL in addition to mere depressive symptomology. In our study, despite being closely related, anxiety contributes to lower HRQoL above and beyond depressive symptomology and motor symptoms, and can be distinguished from both. The interrelation between anxiety, motor symptoms and depression may be due to shared underlying disease mechanisms as well as diagnostic criteria. Considering the impact of anxiety on HRQoL, an integrated, holistic diagnostic approach is needed to identify anxiety in PwPD.

### Electronic supplementary material

Below is the link to the electronic supplementary material.

Supplementary Material 1

## Data Availability

The datasets used and/or analysed during the current study are available from the corresponding author on reasonable request.

## Abbreviations

BAI: Beck Anxiety Inventory
BDI II: Revised version of the Beck Depression Inventory
DSM: Diagnostic and Statistical Manual of Mental Disorders
GAS: Geriatric Anxiety Scale
HADS-A: Hospital Anxiety and Depression Scale
HRQoL: Health-related quality of life
IQR: Interquartile range
MCT: Multimodal Complex Treatment
MDS: Movement Disorder Society
MDS-UPDRS: Movement Disorder Society-sponsored revision of the Unified Parkinson’s Disease Rating Scale
MMSE: Mini-Mental State Examination
NMS-Q: Non-Motor Symptoms Questionnaire
PAS: Parkinson Anxiety Scale
PD: Parkinson’s disease
PDQ-39: Parkinson’s Disease Questionnaire 39
PwPD: people with Parkinson’s disease
SEM: Structural equation model
QoL: Quality of life
