# Supplementary material for: An examination of anxiety and its influence on health-related quality of life in Parkinson’s disease using the geriatric anxiety scale: a cross-sectional study
Source: BMC Geriatr. 2024 Mar 28;24:298. doi: 10.1186/s12877-024-04911-8 (PMC10979635; doi:10.1186/s12877-024-04911-8)
Supplement: Supplementary file 1 — Supplementary Material 1 [file 12877_2024_4911_MOESM1_ESM.pdf]

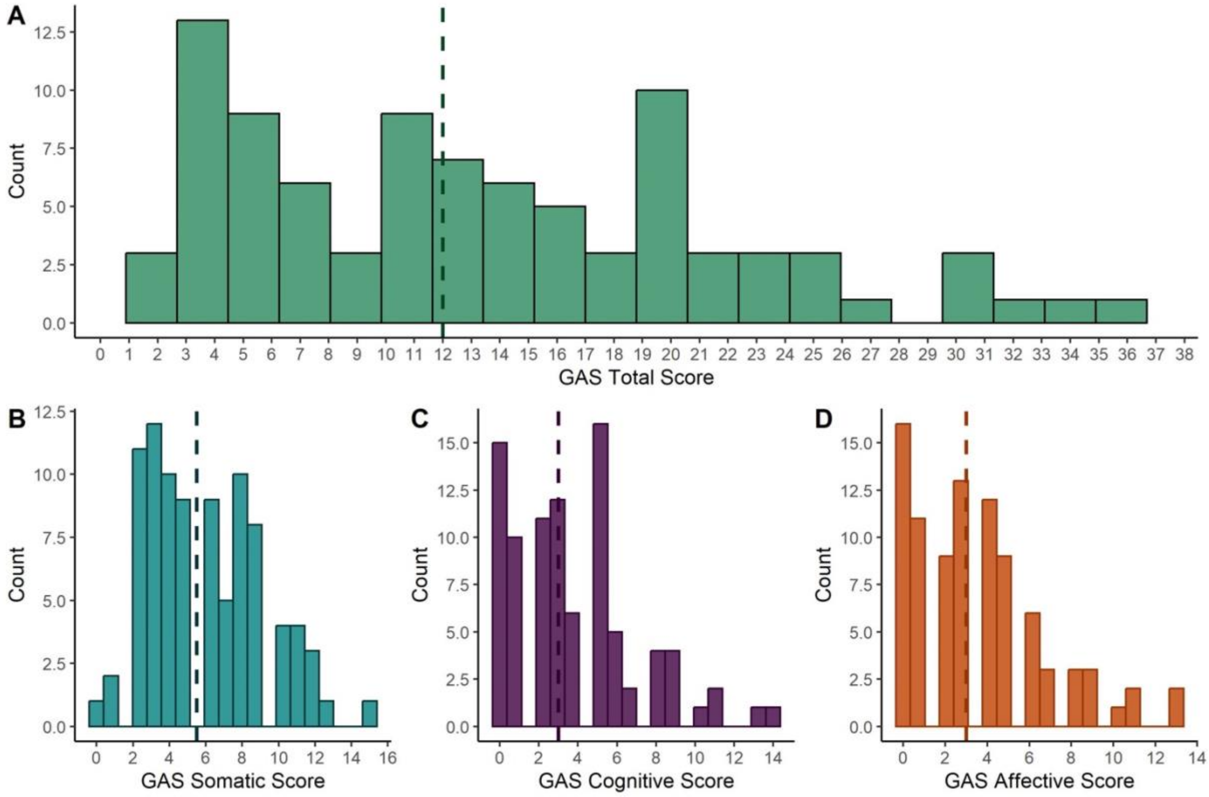

Figure S1. Distribution of the Geriatric Anxiety Scale total score (A) and its subscales (B-D). The broken lines represent the median values.

Table S1: Spearman correlations of the study population.

		GAS	Age	BDI II	MDS-UPDRS III	NMS-Q	MMSE	PDQ-39 SI
GAS	r_s	1.000	0.023	0.622*	0.262*	0.085	-0.083	0.596*
	p	/	0.831	<.001	0.026	0.496	0.434	<.001
Age	r_s	0.023	1.000	0.107	0.174	-0.136	-0.178	0.156
	p	0.831	/	0.338	0.144	0.274	0.092	0.184
BDI II	r_s	0.622*	0.107	1.000	0.273*	0.062	-0.324*	0.447*
	p	<.001	0.338	/	0.027	0.633	0.003	<.001
MDS-UPDRS III	r_s	0.262*	0.174	0.273*	1.000	-0.178	-0.206	0.560*
	p	0.026	0.144	0.027	/	0.189	0.082	<.001
NMS-Q	r_s	0.085	-0.136	0.062	-0.178	1.000	0.028	-0.003
	p	0.496	0.274	0.633	0.189	/	0.825	0.983
MMSE	r_s	-0.083	-0.178	-0.324*	-0.206	0.028	1.000	-0.165
	p	0.434	0.092	0.003	0.082	0.825	/	0.159
PDQ-39 SI	r_s	0.596*	0.156	0.447*	0.560*	-0.003	-0.165	1.000
	p	<.001	0.184	<.001	<.001	0.983	0.159	/

Correlations are given by the Spearman's correlation coefficient r_s. Age: Patients age; BDI II: Revised version of Beck Depression Inventory; GAS: German version of the Geriatric Anxiety Scale; MDS-UPDRS III: Movement Disorder Society-sponsored revision of the Unified Parkinson's Disease Rating Scale part III; MMSE: Mini-Mental State Examination; NMS-Q: Non-Motor Symptoms Questionnaire; PDQ-39 SI: Parkinson's Disease Questionnaire 39 summary index. Significance is indicated by *p<0.05.

Table S2: Impact of the GAS subscales on PDQ-39 summary index.

		B	SD	95% CI lb	95% CI ub	*ß*	t	p
Model 1	(Constant)	-20.296	23.324	-67.474	26.882		-0.870	0.390
	Age	0.302	0.220	-0.144	0.748	0.156	1.371	0.178
	Sex	-3.572	4.096	-11.857	4.714	-0.100	-0.872	0.389
	BDI II	-0.113	0.413	-0.948	0.722	-0.040	-0.273	0.786
	MDS-UPDRS III	0.253	0.131	-0.012	0.517	0.252	1.934	0.060
	NMS-Q	0.174	0.365	-0.565	0.913	0.054	0.477	0.636
	MMSE	0.148	0.594	-1.053	1.349	0.028	0.249	0.805
	Somatic GAS	0.848	0.802	-0.775	2.470	0.158	1.057	0.297
	Cognitive GAS	1.760	1.257	-0.782	4.302	0.230	1.400	0.169
	Affective GAS	2.454	1.419	-0.417	5.325	0.295	1.729	0.092
Model 7	(Constant)	5.641	3.906	-2.226	13.508		1.444	0.156
	MDS-UPDRS III	0.280	0.118	0.042	0.518	0.279	2.371	0.022*
	Cognitive GAS	2.354	1.109	0.120	4.588	0.308	2.123	0.039*
	Affective GAS	2.438	1.139	0.145	4.732	0.293	2.141	0.038*

Values were obtained using multiple linear regression analysis with backward selection to identify the particular GAS subscales that predict HRQoL. Dependent variable: PDQ-39 summary index. Independent variables: Age, sex, BDI II, MDS-UPDRS III, NMS-Q, MMSE, and the somatic, cognitive, and affective GAS subscales. BDI II: Revised version of Beck Depression Inventory; GAS: German version of the Geriatric Anxiety Scale; MDS-UPDRS III: Movement Disorder Society-sponsored revision of the Unified Parkinson's Disease Rating Scale part III; MMSE: Mini-Mental State Examination; NMS-Q: Non-Motor Symptoms Questionnaire, PDQ-39: Parkinson's Disease Questionnaire 39. B: Unstandardized regression coefficient. *ß*: Standardized regression coefficient. CI lb: Lower bound of the confidence interval. CI ub: upper bound of the confidence interval. Significance is indicated by $^*p<0.05$.

Table S3: Model comparison for linear models on the association between a) PDQ-39, GAS and BDI II and b) PDQ-39, GAS and MDS-UPDRS III

A) Association between PDQ-39, GAS and BDI II

Predictor	Est.	CI		p
**Model 1**: PDQ-39 ~ GAS, adjusted R^2 = .396, F(1,72) = 48.94, p < .001				
(Intercept)	13.08	7.30 – 18.85		<0.001
GAS	1.26	0.90 – 1.62		<0.001
**Model 2:** PDQ-39 ~ BDI II, adjusted R^2 = .313, F(1, 65) = 31.06, p < .001				
Intercept	13.51	6.55 – 20.46		< .001
BDI II	1.35	0.86 – 1.83		< .001
**Model 3**: PDQ-39 ~ BDI II + GAS, adjusted R^2 = .440, F (2, 64) = 26.90, p < .001				
Intercept	10.49	4.03 – 16.95		.002
BDI II	0.44	-0.19 – 1.07		.163
GAS	1.07	0.53 – 1.61		< .001
Model Comparison (anova): Model 3 > Model 2 p < .001, Model 3 = Model 1, p = .163				
**Model**	**BIC**	**AIC**	**R^2**	**RMSE**
Model 1	558.0	551.4	.431	14.168
Model 2	570.6	564.0	.313	15.572
Model 3	560.1	552.0	.440	13.953

GAS Subscale Analysis

PDQ-39 ~ GAS affective + BDI II

adjusted R^2 = .356, F(2,64) = 19.23, p < .001, BDI (est = 0.70, p = .063), GAS affective (est = 2.04, p = .024)

PDQ ~ GAS somatic + BDI II

adjusted R^2 = .416, F(2,64) = 24.48, p < .001, BDI (est = 0.88, p = .001), GAS somatic (est = 2.08, p < .001)

PDQ ~ GAS cognitive + BDI II

adjusted R^2 = .417, F(2,64) = 24.56, p < .001, BDI (est = 0.63, p = .040), GAS cognitive (est = 2.39, p < .001)

B) Association between PDQ-39, GAS and MDS-UPDRS III

Predictor	Est.	CI	p
**Model 1:** PDQ-39 ~ GAS, adjusted R^2 = .396, F(1,72) = 48.94, p < .001			
(Intercept)	13.08	7.30 – 18.85	<0.001
GAS	1.26	0.90 – 1.62	<0.001
**Model 2**: PDQ-39 ~ MDS-UPDRS III, adjusted R^2 = .166 F(1,71) = 15.37, p < .001			
Intercept	16.97	9.36 – 24.57	<0.001
MDS-UPDRS III	0.41	0.20 – 0.63	<0.001
**Model 3**: PDQ-39 ~ MDS-UPDRS III + GAS, adjusted R^2 = .476, F(2,70) = 33.70, p < .001			
Intercept	5.56	-1.40 – 12.52	.116
MDS-UPDRS III	0.30	0.12 – 0.47	.001

GAS	1.14	0.79 – 1.48	< .001	
Model Comparison (anova): Model 3 > Model 2 p < .001, Model 3 > Model 1, p < .001				
**Model**	**BIC**	**AIC**	**R^2**	**RMSE**
Model 1	607.3	600.4	0.396	14.39
Model 2	630.8	602.4	0.166	16.62
Model 3	600.2	591.0	0.476	13.21

GAS Subscale Analysis

PDQ-39 ~ GAS affective + MDS-UPDRS III

adjusted R^2 = .403, F(2,70) = 25.25, p < .001, MDS-UPDRS III (est = 0.36, p < .001), GAS affective (est = 2.78, p < .001)

PDQ-39 ~ GAS somatic + MDS-UPDRS III

adjusted R^2 = .379, F(2,70) = 22.94, p < .001, MDS-UPDRS III (est = 0.29, p = .004), GAS somatic (est = 2.60, p < .001)

PDQ-39 ~ GAS cognitive + MDS-UPDRS III

adjusted R^2 = .460, F(2,70) = 31.66, p < .001, MDS-UPDRS III (est = 0.32, p <.001), GAS cognitive (est = 2.84, p < .001)

BDI II: Revised version of Beck Depression Inventory; GAS: German version of the Geriatric Anxiety Scale; MDS-UPDRS: Movement Disorder Society-sponsored revision of the Unified Parkinson's Disease Rating Scale part III; PDQ-39: Parkinson's Disease Questionnaire 39.
